# Comparing improvement ratings and minimal clinically important difference values between patients and physical therapists

**DOI:** 10.7717/peerj.21001

**Published:** 2026-03-23

**Authors:** Yongni Zhang, Yi Zhu, Weimin Li, Yuan Gao, Dongmei Ai, Xin Zhang, Xiaowen Lian, RobRoy L. Martin, Xiongwei Xu

**Affiliations:** 1 Duquesne University, Pittsburgh, PA, United States of America; 2 The Fifth Affiliated Hospital of Zhengzhou University, Zhengzhou, Henan, China; 3 The Second Affiliated Hospital of Hainan Medical University, Haikou, Hainan, China; 4 Zibo Traditional Chinese Medicine Hospital, Zibo, Shandong, China; 5 Nanjing Drum Tower Hospital, Nanjing, Jiangsu, China; 6 Shanghai Yangzhi Rehabilitation Hospital (Shanghai Sunshine Rehabilitation Center), School of Medicine, Tongji University, Shanghai, China; 7 School of Medicine, Tongji University, Shanghai, China; 8 Rehabilitation Hospital Affilated to Fujian University of Traditional Chinese, Fuzhou, Fujian, China; 9 UPMC Center for Sports Medicine, Pittsburgh, PA, United States of America; 10 Kunshan Rehabilitation Hospital, Kunshan, Jiangsu, China

**Keywords:** Patient self-reported outcome measure, PROM, Responsiveness, Psychometric property, Clinical relevance

## Abstract

**Background:**

The minimal clinically important difference (MCID) is most commonly defined using patient-reported assessments of meaningful change. However, MCID values may also be derived from clinician judgments. Patient and physical therapist (PT) ratings of meaningful change may differ due to differences in clinical priorities and observational focus. It remains unclear whether MCID values defined by PTs align with those defined by patients themselves.

**Methods:**

This study aim to determine the agreement in perceived improvement between patients and PTs and compare MCID values for the Simplified Chinese Lower Extremity Functional Scale (SC-LEFS) based on these improvement ratings. Patients aged 18–50 years with non-osteoarthritic lower extremity injuries completed the SC-LEFS at initial assessment and 4-weeks follow-up. After 4-weeks of physical therapy, patients were classified into “improved” and “not improved” groups by using a 7-point global rating of change (GROC) scale. Based on both the patient’s and physical therapist’s independent ratings, patients were separately categorized as “improved” (those reporting being improved or much improved) or “not improved” (those reporting slightly improved, no change, slightly worse, worse, or greatly worse). Separate SC-LEFS MCID values were determined that best distinguished between the two groups for the patient and PT rating. Receiver operator characteristic (ROC) curve analyses were done to define the sensitivity and specificity for these MCID values.

**Results:**

A total of 763 patients with lower extremity injuries were included. Agreement between patients’ and PTs’ rating was substantial (quadratic weighted kappa = 0.8: 95% CI [0.77–0.84]). MCID values of 9.5 were found for both patient report and PT rating, with sensitivity and specificity ranging between of 0.66 and 0.72. The area under the cures (AUC) were 0.74 (95% CI [0.71–0.78]) for both patient report and PT rating.

**Conclusion:**

After 4 weeks of physical therapy, patients and PTs demonstrated agreement in their improvement ratings, and the SC-LEFS MCID thresholds for defining improvement were same for both groups.

## Background

Patient self-reported outcome measures (PROMs) are widely used in clinical and research settings (Zhang et al., 2023a; Zhang et al., 2023b). Psychometric evidence for PROMs, such as reliability and validity, is important to ensure that patients’ health status is being accurately and appropriately represented (Zhang et al., 2023a). In addition, responsiveness refers to the ability of an instrument to detect change over time in the construct being measured (Husted et al., 2000; Mokkink et al., 2010; Terwee et al., 2003; Ueland et al., 2021). Responsiveness can be evaluated using both distribution-based and anchor-based methods (Husted et al., 2000; Jaeschke, Singer & Guyatt, 1989). The minimal clinically important difference (MCID) provides a clinically interpretable threshold for meaningful change and is most commonly determined based on patient-reported assessment (Husted et al., 2000; Jaeschke, Singer & Guyatt, 1989; Terwee et al., 2003; Ueland et al., 2021). However, MCID values may vary by symptom severity, clinical population, and sociodemographic factors. In clinical practice, MCIDs represent meaningful improvement beyond measurement error or statistical significance and are commonly used by physical therapists to evaluate patient progress. However, it remains unclear whether MCID values defined by physical therapists align with those defined by patients.

A MCID value represents a change in score on PROMs that indicates a clinically important change in status (Ueland et al., 2021). The common method used to define MCID is the anchor-based method with the patient’s perspective in their status as the external criterion (Ueland et al., 2021; Zhang et al., 2023a). Patients are typically asked to report changes in their status on an global rating of change (GROC) scale or visual analog scale with range of options that indicate various levels of being “not improved” to “improved”. The responses on this scale determine the MCID values or threshold of change on the PROMs score that corresponds to a meaningful improvement from the patients’ perspective (Ueland et al., 2021; Zhang et al., 2023a). It is unknown if patients’ and physical therapists’ perspective change in status are in agreement and how any differences could potentially effect interpretation of MCID values. Clarifying potential discrepancies between patient- and therapist-derived MCIDs is clinically important, as misalignment may affect goal setting and treatment planning.

Patients and physical therapists may have a different perspective on functional status change. Exploring these differences and their impact on MCID values represent an important step forward in PROMs research. The first purpose of this study is to determine the agreement in perceived improvement between and patients and physical therapists. The second purpose is to compare MCID values for the Simplified Chinese Lower Extremity Functional Scale (SC-LEFS) based on the ratings of improvement from both patients and physical therapists.

### Materials & Methods

### Patients or participants

This study was a prospective cohort study (Level 2), approved by the Institutional Review Board of the Rehabilitation Hospital affiliated with Fujian University of Traditional Chinese Medicine (2021KY-016-01). All subjects provided written informed consent prior to participation. The study was registered with the Chinese Clinical Trial Registry (ChiCTR2100052104). The study included patients aged 18 to 50 years with lower extremity injuries. Patients were excluded if they had weight-bearing restrictions, primary osteoarthritis, neurological disorders, an inability to understand written Chinese, or if they did not complete the four-week physical therapy program. All data was prospectively collected from ten physical therapy clinics in the Republic of China between October 2021 and October 2022. Patients with primary osteoarthritis were excluded at enrollment according to predefined inclusion and exclusion criteria. All participants who met these criteria were included in the analytic dataset, and cases with missing outcome data were excluded prior to statistical analysis.

### Outcome measure

The SC-LEFS is a cross-culturally adapted version of the Lower Extremity Functional Scale (LEFS) used to assess functional status in individuals with lower extremity musculoskeletal injuries (Xu et al., 2020; Zhang et al., 2020; Zhang et al., 2025b; Zhang et al., 2025a; Zhang et al., 2023a). The SC-LEFS has demonstrated evidence of reliability, validity, and responsiveness, supporting its use in various lower extremity injuries (Xu et al., 2020; Zhang et al., 2020; Zhang et al., 2025b; Zhang et al., 2025a). Similar to the original English LEFS, it consists of 20 items, each scored from 0 (extreme difficulty) to 4 (no difficulty). The total score, obtained by summing the individual item scores, ranges from 0 (extreme limitations) to 80 (no functional limitations) (Zhang et al., 2025a). A previous study defined the MCID value for the SC-LEFS in lower extremity injuries after four weeks of treatment, based on patients’ perspectives (Zhang et al., 2025a).

### Procedures

Diagnoses, determined by physicians or physical therapists, were categorized using the International Classification of Diseases, 11th Revision (ICD-11). At the initial assessment and again at the physical therapy four-week follow-up, patients completed the SC-LEFS. At the four-week follow-up, patients and physical therapists were asked to evaluate the functional status change compared to the initial assessment. The questions “How do you feel your functional status has changed since the first time you completed the SC-LEFS?” and “How do you feel your patients’ functional status has changed since the first time they completed the SC-LEFS?” were provided separately to patients and physical therapists, respectively. A seven-point GROC scale was used, with options of greatly worse, worse, slightly worse, no change, slightly improved, improved, or much improved (Zhang et al., 2025a; Zhang et al., 2025b; Zhang, Zang & Martin, 2025). Based on both the patient’s and physical therapist’s independent ratings, patients were separately categorized as “improved” (those reporting being improved or much improved) or “not improved” (those reporting slightly improved, no change, slightly worse, worse, or greatly worse) (Zhang et al., 2025a; Zhang et al., 2025b; Zhang, Zang & Martin, 2025). The rationale for including ‘slightly improved’ in the ‘not improved’ group was to define a more distinct and clinically meaningful improvement threshold (De Vet et al., 2007). Patients received routine physical therapy care as determined by the treating physical therapists at each participating clinic. Treatment was individualized according to the patient’s diagnosis, clinical presentation, and functional limitations and was therefore not standardized across sites or therapists.

### Statistical analyses

Statistical analyses were performed using the IBM SPSS software package (version 27). Quadratic weighted kappa analysis was performed to determine the agreement between patients’ and physical therapists’ ratings of improvement. The strength of agreement for kappa values ranges from almost perfect (0.81 to 1.00), substantial (0.61 to 0.80), moderate (0.41 to 0.60), fair (0.21 to 0.40), to slight (0.00 to 0.20) (McHugh, 2012). Separate SC-LEFS MCID values were identified to best differentiate between improved and not improved groups based on patient and physical therapist assessments. Receiver operating characteristic (ROC) curve analyses were conducted to define the sensitivity and specificity of these MCID values (Glassman et al., 2008; Harris et al., 2017; Katz, Paillard & Ekman, 2015; Nwachukwu et al., 2018). The Youden index was used to determine the MCID change score that provided optimal sensitivity and specificity (Schisterman et al., 2005). The accuracy of this MCID change score was validated by calculating the area under the curve (AUC) with a 95% confidence interval (CI). An AUC that exceeds 0.70 with 95% CI that does not include 0.5 is consider acceptable (Beaton, 2000; Lasko et al., 2005).

## Results

After excluding 11 participants (1%) due to missing data, a final sample of 763 patients was included in the analysis. Demographic characteristics of the study population are presented in Table 1. Musculoskeletal injuries as diagnosed with ICD-11 codes are presented in Table 2. Based on patient self-report, 494 (64.7%) were classified as “improved” and 269 (35.2%) as “not improved”, whereas physical therapist ratings classified 525 (68.8%) as “improved” and 238 (31.1%) as “not improved” (Table 3). The agreement between patient and physical therapist ratings was substantial, with a quadratic weighted kappa value of 0.8 (95% CI [0.77–0.84]). MCID values of 9.5 were identified for both patient reports and physical therapist ratings, with sensitivity and specificity ranging between 0.66 and 0.72. The area under the curve (AUC) was 0.74 (95% CI [0.71–0.78]) for both patient reports and physical therapist ratings (Table 4).

**Table 1:** Demographic information for patients.

	Total sample
N	763
Age (SD) years	32.9 (9.4)
Female	342 (44.8%)
Male	421 (54.2%)
Symptom duration (SD) days	173 (362)
Surgery	251 (32.9%)
Initial Mean LEFS (SD)	49.5 (15.2)
4-week Mean LEFS (SD)	62.7 (12.4)

**Notes.**

SD, Standard deviation.

**Table 2:** Diagnosis information for patients.

Diagnosis (ICD-11 code)	
Dislocation or strain or sprain of joints or ligaments of knee (NC93)	178 (23.3%)
Dislocation or strain or sprain of joints or ligaments at ankle or foot level (ND 14)	146 (19.1%)
Fractures of the hip, thigh and lower leg including ankle and foot (NC72, NC92 and ND13)	143 (18.7%)
Injury of muscle, fascia, tendon or bursa at lower leg level (NC 96)	127 (16.5%)
Certain specified joint disorders of limbs (FA30-FA38)	85 (11.1%)
Injury of muscle, fascia, tendon or bursa at hip or thigh level (NC 76)	75 (9.8%)
Combined injury of bone fracture, ligament and soft tissue (NC72, NC92 ND13, NC93, ND 14, NC76 and NC 96)	9 (0.9%)

**Notes.**

ICD-11, International Classification of Diseases, Eleventh Revision.

**Table 3:** Comparison of patient and physical therapist ratings of improvement.

	Therapist: not improved	Therapist: improved	Total
Patient: not improved	208 (77.3%)	61 (22.7%)	269 (35.2%)
Patient: improved	30 (6.1%)	464 (93.9%)	494 (64.7%)
Total	238 (31.1%)	525 (68.8%)	763 (100%)

**Table 4:** Responsiveness for simplified chinese lower extremity functional scale.

	Minimal clinically important difference value	Specificity (95% CI)	Sensitivity (95% CI)	Area under the curve (95% CI)
Patients report	9.5	0.71 (0.67–0.74)	0.68 (0.64–0.71)	0.75 (0.71–0.78)
Physical therapist report	9.5	0.72 (0.68–0.75)	0.66 (0.62–0.69)	0.74 (0.71–0.78)

**Notes.**

CI, Confidence interval.

## Discussion

This study revealed a substantial agreement between patients’ and physical therapists’ ratings of improvement following a four-week physical therapy program for lower extremity injuries. The MCID values for both patients and physical therapists were 9.5 on the SC-LEFS. This value accurately distinguished between patients who were “improved” from those “not improved” after four weeks of physical therapy. In clinical practice this alignment strengthens the use of the SC-LEFS as an outcome measure for monitoring patient progress and offers a benchmark that reflects both patient experiences and physical therapist evaluations. Although patients and physical therapists may assess “improvement” from different perspectives, this study is the first to show that there is no difference in MCID values between the two groups. This finding builds a foundation for future clinical practice and research, indicating that the MCID value may effectively reflect both the patients’ experiences and the physical therapists’ evaluation.

Although this current study found the same MCID value, there were some differences between patients’ and physical therapists’ ratings of improvement. Physical therapists were more likely to rate their patients improved when compared to the patients’ self-assessments. Specifically, 22.7% patients who rated themselves as “not improved” were rated “improved” by their physical therapists. This discrepancy may be attributed to the physical therapist underestimating the impact that symptoms and impairments have on activity and social participation. Alternately, 6.1% of patients who rated themselves as “improved” were rated as “not improved” by their physical therapists (Table 3). This discrepancy may be potentially explained by patients rating themselves as “improved” as to not disappoint their physical therapists when in reality they were actually not improved.

Although some variations were observed, the differences between patients’ and physical therapists’ ratings on the seven-point GROC scale were small, with quadratic weighted kappa of 0.8, indicating substantial agreement. Quadratic weighted kappa assigns weights to disagreements based on the squared distance between categories, meaning that minor discrepancies have less impact on the overall kappa score (Sim & Wright, 0000). Despite these differences, the final MCID value identified is the same for both groups. This consistency suggests that while individual perceptions may vary slightly, the overall threshold for meaningful improvement is aligned between patients and physical therapists. To apply these values clinically, if a patient initially scores 40 out of 80 on the SC-LEFS, those who score above 49.5 after four weeks of physical therapy are likely to perceive themselves as improved and have their physical therapists also indicate improvement.

In this study, MCID values were determined using anchor-based questions from both patients and physical therapists. Although patients and physical therapists were asked the same anchor-based question, their perspectives on functional status change may be different. Patients’ self-assessments are often deeply influenced by their daily life experiences. Physical therapists, on the other hand, often focus on objective measures and outcomes that align with clinical guidelines and standardized assessments. Discrepancy between the issues that patients and physical therapists consider important have been documented (Lieberman et al., 1996; Martin et al., 2009; Roos, 2001). Notably, differences have been observed between patients and clinicians in how they rated the importance of issues related to symptoms and functional limitations (Martin et al., 2009). Disagreements between patients’ expectations and achieved outcomes have also been reported in the surgical literature, especially among athletes (Ardern et al., 2013; Griffith et al., 2021; Mancuso et al., 2019). However, Shukla et al. (2018) identified patient-physician agreement in outcomes when asking “Compared to before surgery, is your elbow/shoulder better, worse or no different?” with different ratings for worse or better. Differences between patients’ and physical therapists’ perspective and the effect it has on interpreting outcomes may ultimately be context dependent. This study found that although there were some difference differences between the patients’ and physical therapists’ rating of improvement, these differences did not alter the interpretation of change scores on the SC-LEFS scores using a MCID value.

Documenting patient improvement in health status is important for all stakeholders, including patients, providers, policy makers, and payers. A variety of gold-standards can be used to establish benchmarks for patient improvement. Given the high value placed on patients’ perspective when defining outcomes using PROMs, it is helpful to understand physical therapists’ and patients’ perspective of improvement are well aligned, and whether any differences between these perspectives meaningfully affect the interpretation of score changes. To our knowledge this is the first study to compare the agreement between physical therapists and patients in rating of improvement after physical therapy. This study is also unique in that fact it compared MCID values determined separately for patient and physical therapists to define improvement after 4 weeks of physical therapy. While this study was done on the SC-LEFS for those with lower extremity musculoskeletal injuries, it will be of interest to see if similar findings can be achieved with other populations and PROMs.

### Limitation

The findings of this study are limited by the context and methodology used. The results of this study pertain to Chinese speaking individuals with lower extremity musculoskeletal injuries. Cultural factors may also influence patient–clinician interactions and self-reported outcomes. In addition, a larger proportion of participants were classified as improved, which may have influenced the estimation of the MCID threshold and the kappa agreement. Additionally, the MCID values defined in this study apply only to the SC-LEFS and over a 4-week physical therapy treatment interval. The study also excluded patients with primary osteoarthritis and therefore potentially less active individuals. Data on physical therapist characteristics were not collected. Therefore, variability in clinician-derived judgments of meaningful change could not be examined. All these limitations effect the generalizability of the findings.

## Conclusion

This study found patients and physical therapists agreed on improvement ratings after 4-weeks of physical therapy in lower extremity musculoskeletal patients. MCID values to define improvement on SC-LEFS after 4-weeks of physical therapy were the same for both patients and physical therapists’ perspective. This finding builds a foundation for future clinical practice and research, suggesting that the MCID value can effectively bridge the gap between the differing perspectives of patients and physical therapists regarding functional improvement.

## Supplemental Information

10.7717/peerj.21001/supp-1 Supplemental Information 1 STROBE Checklist

10.7717/peerj.21001/supp-2 Supplemental Information 2 Dataset

## Additional Information and Declarations

### Data Availability

The following information was supplied regarding data availability: The raw measurements are available in the Supplemental File.

### Clinical Trial Registration

The following information was supplied regarding Clinical Trial registration: ChiCTR2100052104.
